# Schools as spaces for in/exclusion of young Mainland Chinese students and families in Hong Kong

**DOI:** 10.1186/s40878-021-00269-7

**Published:** 2021-12-16

**Authors:** Maggi W. H. Leung, Johanna L. Waters, Yutin Ki

**Affiliations:** 1 Department of Geography, Planning and International Development Studies, University of Amsterdam, Nieuwe Achtergracht 166, 1018 WV Amsterdam, The Netherlands; 2 Department of Geography, University College London, 26 Bedford Way, London, WC1H 0AP UK; 3 Hong Kong Association for Transport Education, Box 35162, King’s Road Post Office, North Point, Hong Kong SAR, China

**Keywords:** School, Children, Border, Migration, Inclusion, Organisation, Hong Kong, Shenzhen, China

## Abstract

Around 30,000 children living in Shenzhen, Mainland China cross the border to Hong Kong to attend school every day. This paper focuses on the school as a key meso-level organisation that mediates macro-level policies and micro-level everyday life experiences among these children and their families. We advocate a relational, spatial perspective, conceptualising schools as webs of intersecting physical, social and digital spaces, where differences between the “locals” and “others” are played out, negotiated and (re)produced, and in turn giving rise to specific (and understudied) geographies of in/exclusion. Drawing on our qualitative research, we offer a close reading of three exemplary school spaces: (i) the physical classroom and school grounds, (ii) the digital classroom, and (iii) at the school gate. Our findings demonstrate the complex and at times contradictory ways in which “the school” is a place of both inclusion and exclusion. It is a dynamic and power-traversed space where social differences between the “locals” and the “others” are played out, contested and redefined continuously.

## Introduction

Tens of thousands of children (27,000 as of May 2020, cited in RTHK, 2020) who live in Mainland China, predominately in Shenzhen, cross the border daily to attend school in Hong Kong.^1^ Being “hypermobile” for schooling, these cross-border students (CBS) embody “educational mobilities” (Lipura & Collins, 2020). Yet, mobile children have, to date, been rather sidelined in the scholarship. Research on educational mobilities has focused instead on international students pursing higher education (Brooks & Waters, 2011; Finn & Holton, 2019; Waters & Brooks, 2021). The prototype “international students” in this body of work are young adults who make infrequent trips to their place of study where they establish a new home and social life. Compared to them, CBS experience education mobilities very differently. Their pursuit of a “better education” necessitates *quotidian* border crossings and a commute that might take a few hours a day. This distinct spatio-temporality intensifies the role of the border, the state and the journey (Waters & Leung, 2019). Furthermore, it underlines the importance of the school as *the* key place where CBS derive most of their daily social experiences. For migrant children, like the CBS in our research, the school is far from “just” a place of learning. It is also a central site where socialisation with “locals” takes place. At school, these children build relationships with their peers, teachers and other staff, as well as other parents. As such, the school is a key place where social identities and power relations are (re)produced and contested and in/exclusion is entrenched (Holt, 2003).

Yet, the school has remained relatively underexamined in extant literature on education mobilities (but see Devine & Kelly, 2006; Lesar et al., 2006; Crush & Tawodzera, 2014; Zhang, 2018). Our paper contributes to filling this gap. As a part of this special issue on the role of organisations in shaping migration and inclusion, we examine the school as a meso-level organisation that links micro- and macro-level political and social processes. Hereby, we echo the arguments by Pries (2008) regarding the usefulness of the scale of organisations in studying processes in transnational social contexts as they provide a micro–macro-link between everyday life and social institutions. More specifically, we put forth a relational, spatial perspective in our study of the role of schools in (co)producing geographies of in/exclusion. We engage spatialities at two levels. First, we examine how the school as a meso-level organisation is (i) linked horizontally in a network of other players within and beyond the education system in producing cross-border mobility, and (ii) embedded vertically between macro-level policies (in particular those regulating immigration and education) and micro-level institutions (such as the family). Second, we conceptualise schools as a web of physical, social and digital spaces where differences between “locals” and the “others” are played out, negotiated and (re)produced, and in turn give rise to specific geographies of in/exclusion.

Our focus on in/exclusion processes contributes to critical perspectives on educational mobilities by highlighting their relational—as opposed to the common individualistic framing (Holdsworth, 2013)—and often “dysfunctional” nature (Waters, 2015). We also respond to Thiem’s (2009) early call for more “outward-looking geographies of education”, through our focus on the “various political, cultural, and economic projects pursued through [education’s] content, governing structures, or modes of distribution” (p. 168). In our analysis, we make links between education mobility and broader identity politics, which play an important role in charting the geographies of in/exclusion in the Shenzhen–Hong Kong transborder region (e.g. Xu, 2017).

### School as an organisation for in/exclusion in migration contexts

While the role of the school is less examined in education mobilities research, its importance in cultural reproduction has been a core topic in sociology of education. According to Bourdieu (1977), schools are major social institutions, reproducing cultural and social (in)equities across generations. Schools are used to impart cultural ideas that underpin and uphold the privileged position of the dominant or upper class. Research has repeatedly shown how school systems are more than often institutions perpetuating inequalities, in spite of their egalitarian ethos. In a recent study, for instance, Domina et al. (2017) conclude from their analysis of schools in contemporary USA that they function as “sorting machines”, creating categories that serve as the foundation of later life inequalities.

Schools, and the education system at large, are tightly connected to the economic, social, cultural and political spheres of society. Positioning schools in the broader institutional landscape is important as what happens in the classroom and school is affected by processes and debates in other domains (Collins & Coleman, 2008; Collins 2009). Certainly, schools function with diverse levels of autonomy, often depending on the governance relation with funders, be they the state, church, private corporations, foundations and parents' associations. Publicly funded schools, in particular, are often assumed to be subject to the state’s interests to a large extent. Research in critical education studies has offered useful insights into the role of public education as an institution in achieving the state’s objectives (Mitchell, 2001). Drawing on research predominantly conducted in the USA (Henry, 2020)—our research moves away from this geographical focus - this line of work reveals how education has functioned as a state instrument to advance its economic, socio-cultural and political (producing “ideal citizens” and supporting nation-building) objectives (Mitchell, 2001; Veugelers & Zijlstra, 2004), as well as military (Nguyen, 2014) and security agendas (Lizotte & Nguyen, 2020). Yet, it would be simplistic to think of state-funded schools as docile factories promoting geopolitical and socio-cultural ideologies. Rather, schools (and individuals within them) also exercise agency in carrying out, remaking and contesting states’ agendas (Lizotte & Nguyen, 2020). Focusing on the spaces that make up the school allows us to map out how agency is played out. As we will illustrate from our research, organisational rules and cultures are implemented on the ground by teachers and other staff, who often exercise their discretion when acting in this regard.

This paper draws on extant scholarship on in/exclusion of migrant children at schools in different contexts. In Europe, earlier work includes Devine and Kelly’s (2006) study on the processes of inclusion and exclusion in multi-ethnic primary schools in Ireland. In another example of work in this vein, Lesar et al. (2006) focus on the situation of Roma/Gypsies and migrant children from former Yugoslavia at schools in Slovenia. Since 2015 when large number of refugees arrived in Europe, a wealth of research has examined the policies and practices in relation to the integration of refugee children. Focusing on a policy level, Crul et al. (2019), for instance, compare the effect of education policies and school systems in in/exclusion of Syrian refugee children in Sweden, Germany, Greece, Lebanon and Turkey. Other work has brought forth the daily experiences of children. McIntyre and Hall (2020), for example, chart the barriers to the inclusion of refugee and asylum-seeking children in schools in England, while Çelik and İçduygu (2019) analyse the circumstances among Syrian refugee children in different kinds of schools in Turkey. Furthermore, the experiences of refugee and migrant children in Africa have also attracted scholarly attention. Examining integration policy in Kenya, Bellino and Dryden-Peterson (2019), draw attention to the limit of educational “integration” within refugee camps and their long-term inclusion in host societies. Other research highlights the issue of migrant discrimination and exclusion at schools. Crush and Tawodzera (2014), for instance, highlight  the experiences of  Zimbabwean migrant children in South African schools.

Issues of in/exclusion among *internal* migrants have also been investigated, especially in the Chinese context. Existing research has documented the experiences of in/exclusion among rural migrant children in the cities (e.g. Li & Jiang, 2018; Zhang, 2018). Our research brings a further level of complexity to this research field. As opposed to the rural migrants who live and go to school in cities in China, CBS in our study move across a “hard” border twice daily for school. Yet similar to the rural migrant children, they do not only cross an administrative border, but one that also demarcates socio-cultural differences, as we will discuss below.

Logically, the findings and implications of the literature reviewed above are diverse and highly contextualised. As a body of work, they illustrate the tension between policy objectives of integration, that are operationalised mostly through access to education, and social inclusion. Attending school in the host society does not per se mean social inclusion for migrant children. Integrating migrant children in education often means supporting or demanding them to fit into the local system, with the goal of playing down or reducing differences and diversities. This intriguing tension between integration, inclusion and diversity at schools is explored in this paper. Furthermore, we seek to unpack the school, which has mostly been represented as one unit within the literature discussed. We unpack it by mapping out the diverse but intersecting spatialities that constitute it.

### Research context: the Shenzhen–Hong Kong cross-border schooling phenomenon

The children in our study commute between Shenzhen and Hong Kong for school. Though located in the same “one-country”, Shenzhen and Hong Kong are separated by a hard border, similar to an international one, guarded by strict immigration and custom controls. This peculiar situation has to do with the special geopolitical status of Hong Kong. When the former British colony was returned to China in 1997, Hong Kong became a Special Administrative Region. The return of sovereignty was arranged with the principle of “One Country, Two Systems”. Under this framework, Hong Kong was given semi-sovereignty for 50 years after the handover and guaranteed a high degree of autonomy, the rights to retain its capitalist system, independent judiciary and rule of law, free trade and freedom of speech. The unusual legal construct required a clear boundary to exist between the two territories. The border demarcates the divide between two distinct, and yet highly interlinked sets of political, social and economic systems.

The difference between the education systems in Hong Kong and Mainland China and their (perceived) position in the broader international educational context have motivated mobility of a diverse nature. Children and young people move across the border for education and training at different levels (see Lam, 2006; Te, 2020; Xu, 2017, 2019 for research on such cross-border mobilities for higher education).

The focus of our research is CBS, who are mostly children of cross-border marriages between Mainland Chinese and Hong Kong citizens. While the Shenzhen–Hong Kong region has historically been closely linked, cross-border marriages between Hong Kong residents (predominantly men) and Mainland Chinese (predominantly women) have increased substantially with the rise of economic integration and social interactions between Hong Kong and Mainland China since 1970s (Chee, 2017; Lin & Ma, 2008). To migrate formally to Hong Kong, Mainland wives of Hong Kong men often have to wait for four to eight years for a permit. It is estimate that as many as 100,000 cross-border families live in such a state of separation (Society for Community Organisation, 2019). Many of these women use a temporary visitor visa to give birth to their children while waiting for their immigration permit. Children born to these couples are referred to as “one not” children, as one of the parents does not have residency in Hong Kong. Being born in Hong Kong gives these children the right of abode, with which they have access to various social benefits in Hong Kong, including subsidised kindergarten education and 12-year free and compulsory primary and secondary education in the state (public) school system. Many of these children live with their mother in Mainland China while the father works and resides mainly in Hong Kong. This gives rise to the cross-border schooling phenomenon. Many cross-border families remain in Mainland China after the attainment of the immigration permit for the mothers. Lower cost of living, more affordable housing, family and friendship relations on the Mainland are the main reasons for this decision, which also means continuation of the cumbersome commute for the children. In addition, there is also a large group of children whose parents are both Mainland Chinese citizens (so-called “double not”). Since a change of legislation in 2001, all children born in Hong Kong have been entitled to permanent Hong Kong residency, irrespective of their parents’ citizenship. This resulted in a rise of “double not” children engaging in cross-border schooling. Finally, some CBS are born to two Hong Kong residents, both living in China while the children commute to school daily.

Cross-border families are not a homogenous group. They come, for example, from a mix of socio-economic backgrounds. Among “double not” families, many were originally from middle class backgrounds. However, as the market in birth tourism developed, more families from working-class and rural backgrounds have alsosought  Hong Kong residency for the children (Chan & Ngan, 2018; Chee, 2017). Many of these families exhausted much of their savings for the birth of their child in Hong Kong, since tourists do not have access to subsidised health services there. Some of these families have also invested in a new residence closer to Hong Kong when their children begin their cross-border commute.

Depending on the background of the parents, in particular the mother, CBS growing up in Shenzhen may speak Cantonese, the predominant Chinese language in Hong Kong, though they might lack a Hong Kong accent or vocabulary. Others might speak other forms of Chinese (“dialects”) such as Mandarin, Hakka or Teochew. Compared to Hong Kong where the education system still exhibits markers of British colonialism, English knowledge is more limited in Mainland China especially among lower-income families.

**Fig. 1:**
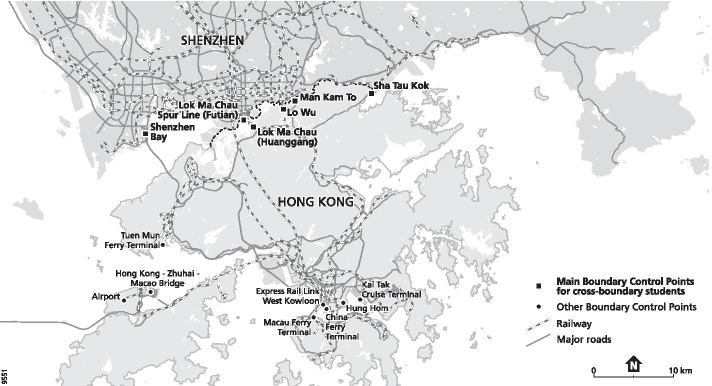
The Shenzhen–Hong Kong border

The vast majority of CBS attend Hong Kong public schools in the North District close to the border (Fig. 1). Depending on the children’s age and the relative location of their home and school, they commute in diverse ways—alone or with company, with or without transit, and with different means of transportation. Few, even among very young children, are accompanied by their parents because of work and other commitments in China and, in many cases, the parents lack the required permit to travel daily across the border. While younger children mostly travel in groups by school bus and/or on trains with caretakers (referred to as “nannies”), older school children travel alone or with their schoolmates by public transportation (Leung & Waters, 2021). In any case, they need to go through four control points to exit China and enter Hong Kong in the morning and return home in the afternoon. Each crossing involves border control by officials from both sides. Even though immigration control has been simplified by both the Hong Kong and Shenzhen governments with the use of dedicated counters, e-channels and “on-board” (on bus) clearance, it sometimes entails a few hours of travelling daily. Although these cross-border trips are routinised (Waters & Leung, 2019), parents whom we interviewed often described these trips as “troublesome” and “exhausting”. The outbreak of the COVID-19 pandemic underlines the restrictive nature of the border. CBS were the first group to be blocked from school as a result of the closure of the border and quarantine rules, before school closure was implemented in Hong Kong. Fluctuations of the pandemic situation and recurrent changes in border controls placed immense stress on CBS children and their parents (Takungpao, 2020).

Over the years, there have been numerous (social) media reports and a relatively small body of academic literature (mainly in social work and education studies) that highlight the challenges these long-term and long-distance commutes pose to the children and their families, who are commonly seen as “trapped” in mobilities (Chee, 2012, 2017). Many studies have highlighted the issue of children’s social exclusion. There are a number of reasons given for this. CBS are often seen to be disadvantaged because of their long commutes, usually relatively low socio-economic status, and difficulties (at first) in mastering Cantonese, written traditional Chinese characters (as opposed to simplified characters used on the Mainland) and English that are the mediums of education in Hong Kong. These factors constrain these children from fully participating in and benefiting from school life, which in turn limits their full integration and social upward mobility (Chan & Kabir, 2014; Chan & Ngan, 2018; Chiu & Choi, 2019; Equal Opportunities Commission, 2019; Yuen, 2011). Little is known, however, about how in/exclusion is played out at schools. This paper illustrates some of these processes.

## Methodology

Our analysis is based on qualitative research that examines parents’ motivations and experiences in sending their children to Hong Kong for school. In addition, perspectives of teachers and social workers who have worked directly with these children were also collected. Physical fieldwork in Shenzhen, Hong Kong and at the border was conducted in 2018 and 2019, supplemented with follow-up interviews, policy and media analyses to track the first impact of the COVID-19 pandemic on CBS, their families, schools and teachers.

The majority of our field data are derived from interviews and observation. Potential interviewees were approached through WeChat, the Chinese messaging, social media and mobile payment app (with the parents) and personal contacts (with teachers and social workers). In total, we interviewed—in some cases with follow-ups—12 families (17 mothers and fathers), a university student (early 20 s), a high-school teenager (16 years-old) and two 6th-graders (12 years-old) who had crossed the border for school/university for years, a grandfather of CBS, a former school principal, five teachers, one school social worker, one government officer working at Shenzhen Customs and one staff member at a tutoring centre near one of the main border crossings. While most of the interviews were face-to-face, some of the conversations were conducted over WeChat or by phone. We also conducted observation at the border crossings and followed along the school journeys of these children and visited related social service centres in Shenzhen. In addition, we conducted an in-depth content analysis of relevant policies, news and social media coverage of the phenomenon. We followed the discussion on two WeChat groups dedicated to cross-border families. Each of the groups had around 400 members at the time of our research. The online groups provided valuable insights into parents’ perceptions, concerns and experiences. Approval for this research was granted by the University of Oxford Central University Ethics Committee.

### Why the pain? Aspirations for better schooling

Confirming previous studies (Chan & Kabir, 2014; Chiu & Choi, 2019; Equal Opportunities Commission, 2019; Yuen, 2011), we have found that a “better education” is the primary motivation for Mainland parents to send their children to school in Hong Kong. Their decision is based on a comparison between the two education systems across the Shenzhen–Hong Kong border. Our interviewees are drawn to the Hong Kong school for its English-language learning environment, open and equitable teacher-student relations, smaller class sizes and an ‘international’ perspective. As Burrow (2017) concludes in his work, going to a Hong Kong school is seen as “a stepping-stone to a better future” (p. 18).

It is important to note that this decision is not only driven by a *desire* for better education; for some it is also a *need*, i.e. the only way to attain an affordable school place. This has to do with the Chinese household registration (*hukou*) system that identifies a person as a permanent resident of a particular location in China. *Hukou* is the defining factor for social rights entitlements including state-funded education. According to the Chinese law, individuals cannot simultaneously possess a *hukou* in Mainland China and right of abode in Hong Kong. The Hong Kong school is, therefore, the only option for those unable  to pay a hefty fine charged by the state for violating the one-child, and late two-child, policy (of RMB 200,000, c.a. 26,000 Euro as reported by DY, mother of a CBS, interviewed in 2018) or expensive school fees charged by private or international schools.

Since the rapid rise in the number of CBS in Hong Kong in 2006, 5 years after the change of legislation in 2001, a series of adjustments has been made to the school systems on both side of the border. An increasing number of government-funded schools in Guangdong Province, where Shenzhen is located, were opened up to these children. Under this policy, Hong Kong children of Mainland parents can apply for these public primary schools in Guangdong through a points system. Yet, the number of places is limited. Priority is given to parents with local *hukou* and those who live in the districts where their targeted schools are located. According to a survey conducted in 2017, only 9% of the 1077 participating Shenzhen-based parents considered transferring their Hong Kong-born children to Mainland public schools (Su, 2017). Many parents were worried that their children might not be able to get into one of the more popular public schools in Shenzhen (Cheung & Zhao, 2017) and they therefore opted for the “better” education available to their children in Hong Kong.

In addition, the Hong Kong school system has moved part of their teaching over the border. Since 2008, the Hong Kong and Shenzhen Education Bureaus have jointly implemented a scheme of schools/classes for Hong Kong children in Shenzhen so that they can attend kindergarten and primary school on the Mainland and be transited smoothly into secondary school education in Hong Kong. The learning programme of the schools/classes for Hong Kong children mainly follows the Hong Kong curriculum. Eligible primary six (last year of primary education) pupils can join the Hong Kong Secondary School Places Allocation System and be allocated subsidised secondary school places in Hong Kong. These schools are, however, private schools that charge higher fees than Chinese public schools. We have also found that for many parents who are able to pay these fees, cross-border schooling is the preferred option, as it enables their children to gain the “real” Hong Kong schooling experience. We asked our interviewees about this. Their responses are characterised by calculative comparisons between the two school systems that are fused with stereotypes and display a distinct hierarchy. LF (Mainland mother of two children, interviewed in March 2018) shared her “calculation”:

My daughter went to a “Hong Kong class” in a kindergarten here in Shenzhen. It has a Hong Kong owner. Here in the neighbourhood, there are about a thousand of these Hong Kong children. So these “Hong Kong classes” are catered for them. They use teaching materials from Hong Kong, but the teachers are local [Mainland Chinese]… I noticed some differences. The Hong Kong education model was indeed very good, but because the staff was local, the “taste” changed. In Hong Kong, teachers teach with their heart. Here, it’s more like, teachers show their love in front of the parents, but once they are gone, the teachers change their face. I know that because I went there often. I observed the teachers, how they had ice-cold faces when parents were not around. I think it is a matter of “human quality” (*suzhi*).^2^ You cannot change that in the short run.

Parents also compared other aspects of the Shenzhen and Hong Kong school systems. SY (Mainland mother of two children, husband Hong Kong resident, interviewed in March 2018) provided us with her list of comparisons:

I could write a book about it. Advantages: first, it is cheap in Hong Kong. I spent around 2,000 HKD one semester [210 Euro]. In Shenzhen, it normally takes RMB 3,000 per month [380 Euro]. Second, in Hong Kong, I think the teachers won’t beat my son. In Shenzhen, I worry that my kid might be beaten (by teachers). Third, Hong Kong has many English-native speakers, and it is good for him to learn English.

SY also shared with us her vague plan to send her son to study overseas. She considered that a Hong Kong passport, English proficiency and an international outlook—all expected to come with the “better” Hong Kong education—would make her dream more reachable. In parents’ imaginaries, therefore, the Hong Kong school is superior because of its organisational culture (such as politeness, teaching with ‘heart’), lower costs and also its relative position in the global education system. The Hong Kong school is seen as a “promising place” for their children’s futures, which is worth the pain that transborder schooling entails. The paradox (i.e. giving their children a good education and promising future, yet putting them through tiring commutes for years) illustrates the power of social imaginary. As conceptualised by Taylor (2004), social imaginaries denote “the common understanding that makes possible common practices and a widely shared sense of legitimacy (p. 23)”. The distinct self- and common understandings, practices and horizons of common expectations give these CBS parents and families a sense of shared group life (Tsao et al., 2018). This sense of community and mutual support can be observed in the WeChat groups that we followed.

Our interview data highlight parents' desire and need for a school place in Hong Kong. It is, however, necessary to emphasise the symbiotic relation between CBS and many schools that are also *in need* of students. Due to persistent below-replacement birth rates in Hong Kong and the reluctance of the government to invest in small-class education, kindergartens, primary and more recently secondary schools are increasingly facing the risk of closure. For kindergartens and schools located close to the border, CBS are attractive “clients”. In order to attract pupils, schools actively produce the aspirations and practices associated with this transborder education field, both materially and discursively. Embedded in a dynamic mobility industry, schools work side by side and sometimes in collaboration with other service providers such as bus companies, daycare and tutoring and boarding house services in accommodating the needs of CBS (Leung & Waters, 2021). The schools’ efforts in (re)producing the social imageries and practice of the transborder education field can be observed in their regular promotion at residential complexes where many of the cross-border families live, on their websites, via social media and at education information events such as the Hong Kong Education Fair for Cross-Border Students. Held in Shenzhen, this annual fair provides interested families with information about the Hong Kong education system in general and gives space to schools and other service providers (e.g. bus companies, tutoring centres) to market their offers (Liu, 2012). As the socio-demographics of the CBS change, this transborder education (business) network has also evolved in its structure and offers.

These schools promote themselves as inclusive organisations, giving extra attention to the special needs of CBS arising from their transborder life. In particular, their demand for safe and efficient transport arrangements, language (Cantonese and English) support and extra-curricular activities are made particular “selling points”. Some schools provide free shuttle services from and to the border. Catering for families who have tight budgets, some schools offer free-of-charge lending services for school books, uniforms and computers. A few kindergartens have opened extra-curricular activity centres in Shenzhen to serve their pupils on the weekends. These centres provide courses in English language, music instruments, drawing etc., which are common after-school or weekend activities for Hong Kong children, delivered in a “Hong Kong style”. Since CBS hurry to return home after school, it is very difficult to squeeze in time for these extra-curricular activities on weekdays whilst commuting to and from Hong Kong. By locating these offers on the Mainland, schools help satisfy, and (re)produce, the desire of CBS parents to replicate an all-round Hong Kong education for their children. While it is not (yet) a common practice for kindergartens and schools to run their own extra-curricular activity centres in Shenzhen, numerous commercial “Hong Kong style” tutoring centres have been providing similar services. As such, a kind of integration is being pursued in a re-territorialised, transborder manner. In a paradoxical way, by learning and playing in an exclusive bubble (away from their peers in Hong Kong and other children in Shenzhen), CBS are being raised, distinctively and separately, as a kind of “Hong Kong children” in transborder space.

In the following, we will take a closer look at the schools in Hong Kong. By charting the processes on the ground, we bring forth the role of the school in mediating macro-level institutional policy directives and micro-level lived experiences among the students, parents and school staff. Our close-up analysis helps to map out “the school” as an intersecting physical, social and digital space where differences are (re)produced and in/exclusion is negotiated (see Brooks & Waters, 2017).

### Schools in the co-production of differences and in/exclusion

In branding Hong Kong as Asia’s World City, its higher education sector (in particular) emphasises ‘diversity’ and ‘internationalism’ (Hong Kong Special Administrative Region Government, 2018). In stark contrast to this, pre-university public education for newcomers and ethnic minorities (mostly of Indian, Pakistani and Nepalese background) is based on an ‘assimilationist’ approach (Yuen, 2011). Education policies and practices, as well as related support programmes aim predominantly to help “non-local” children and youngsters—referring to those of lower-income, non-Cantonese background in policy and public narratives—become “one of us”. Under such policy direction, knowledge of languages used in local schools (i.e. Cantonese and English) and the “Hong Kong way of doing things” are at the core in defining the goals and practices of inclusion in the school systems. Diversity in language and ethno-culture is seen as a challenge to the smooth functioning of public schools, as opposed to international schools that are reserved for those who can pay high fees.Yet, the implementation of these macro-level directives on the ground is by no means straight-forward. In the following, we zoom into some key places and space where diverse identities, belongings and, in turn, in/exclusion are played out, negotiated and (co)produced.

#### Classrooms and school grounds

Language plays an important role in in/exclusive processes in this transborder education field (Gu & Tong, 2012). Officially, CBS students must conform and adapt to the use of Cantonese (written in traditional Chinese characters) and English in class, except in Mandarin Chinese (as second language) lessons. The enforcement is, however, not always strict. Here, the role of teachers in mediating the macro-level policy agenda and micro-level classroom life is important. Due to the increase in the number of CBS over the past 15 years, some teachers have adjusted their demand for “speaking like us” in the classroom. Teacher A, one of our interviewees, who has grown up near the border, and had 15 years of teaching experience in North District, accounted for the more relaxed practice regarding the use of language (interviewed in 2018 in Hong Kong):

Especially earlier, some colleagues found it unacceptable that CBS speak Mandarin in the classroom. If students spoke, they had to do so either in Cantonese or English. I think [said with a giggle] the students nowadays could file a racial discrimination complaint [if the teacher insists].

Certainly, teachers are a diverse group. The extent to which teachers will implement the “language requirement” depends on a number of factors, including: the organisational culture of their school, leadership style and demands of the principal and school board, and the teachers’ own pedagogic orientation and personal views. Shielded in a way by the classroom walls, teachers can use their own discretion when conducting classroom activities and setting and enforcing rules that optimize teaching and learning. Interesting here is also our interviewee’s reference to the Hong Kong Race Discrimination Ordinance. Enacted in 2008, the ordinance protects individuals from racial discrimination or harassment. While our interviewee made the comment in a semi-joking way (without referring to any specific complaints per se), it does show how contestations of differences and processes of in/exclusion at school are embedded in a broader and changing institutional landscape.

The increase in number of CBS has also made easier for the students to speak their language, perform their identity and forge a sense of belonging among themselves. Teacher A continued with his observation that CBS no longer need to fit in the Hong Kong mainstream culture:

When there are more and more CBSs, they start to form a group themselves, speaking Mandarin, talking about Mainland media or topics. They find it good enough to hang around with their CBS friends at school. Whereas ten years ago, when there were fewer CBS, those students felt that they needed to learn and fit into Hong Kong culture.

It is not surprising that CBS develop their “bubble”; in part this is rather “natural” considering that they spend a long time each day together, on the way to school and back home. By forming their own social groups, CBS contest the integration politics in the schools and exclusive tendencies in the broader society (Equal Opportunities Commission, 2019). CBS’ making of their in-group as a “safe space” does not necessarily signal a failure in integration. All our teacher interviewees emphasised that CBS also interact regularly with local Hong Kong students. As children grow and gain fluency in Cantonese, they become more integrated in the local social environment. This shows the fluid and evolving relationship between inclusion and exclusion. Rather than opposites, they are relational processes that can take place in parallel, intersecting and evolving over time.

Schools are not only spaces of classroom learning. Because of the long-time commute, many CBS cannot take part in extra-curricular activities (such as joining a school sports team). Teacher B, who serves as one of the core staff members for organising extra-curriculum activities in his school, regrets the difficulty in engaging CBS in team sports fully:

For any sports training, for example, durability and perseverance are important, or even essential, to have any quality improvement. For CBS, it is difficult. I am a coach, and there are around three to four CBS out of 20 students in my team. They often say they need to leave early, before training finishes. But I cannot reject them from joining the team because they are CBS. And during sports training, the best part is usually at the last part, no matter regarding team-building or for the training of perseverance. If one just joins the earlier or the middle part, the player can just learn the skills, but would lack the feeling of team-work that comes when the team completes the whole training. Because of their lack of time, CBS would feel less being part of a team in sports, I would say. And CBS generally see these sports as leisure rather than a team spirit-building exercise, and do not share the drive in winning the tournament. So, it is hard for CBS to be “professional” in school team’s sports. Worse still, not many CBS even join the school’s sports team at all.

To create an inclusive space, this teacher allows CBS to join the sports team even though they regularly miss important parts of the training. Here, we see how CBS, the schools and their teachers constitute each other’s identities in a dialectic way. It is through on-going negotiations that (idealised) elements of this transborder education field, e.g. the “Hong Kong school”, its “values”, “good teachers” and the “students” are (re)produced and redefined.

#### Digital classrooms

E-learning plays an important role at school in Hong Kong and China. Children are exposed to e-learning early on. Most primary schools and even kindergarten integrate digital technology in their curriculum. This does not, however, mean that all children are equally equipped to access and take part in these digital classrooms. Globally, technology has not enhanced educational equality (Kim, 2018). Inequalities have been found in access, but also students’ ability to use and create with ICT technology. All these contribute to the in/exclusion of specific groups of students. Digital divides are found along lines of socioeconomic status, gender, age, geographic location etc. (Lembani et al., 2020; Milakovich & Wise, 2019; Ritzhaupt et al., 2020). The COVID-19 pandemic has exacerbated the digital inequalities worldwide (Azubuike et al., 2020; Lai & Widmar, 2020). These features of digital inequalities are also observable in our case. CBS from lower-income background are often hindered because of the lack of access to necessary infrastructure, such as hardware and high-speed internet, or technical and other support from their families. Social workers and teachers have long expressed concern about this digital divide, especially when the digital classrooms have become an increasingly important space of learning and social contacts, hence mediating in/exclusion and in the longer-term regulate social mobility. This trend has intensified during the COVID-19 pandemic (Chan, 2020a, 2020b).

In the Chinese context, in/exclusion in the digital space is not only socio-economic and technical, but also a political issue. In Mainland China, certain domain names are heavily controlled and users are often blocked from accessing particular websites. These include websites and Apps popularly used by teachers in Hong Kong schools such as Google, YouTube, Wikipedia, WhatsApp and Zoom. Not being able to access teaching materials and instructions is a recurrent challenge for many CBS. The mismatch between the two digital spaces across the Hong Kong-Shenzhen border is a constant hassle for many CBS, their parents and teachers. Teacher B, whom we interviewed during the COVID-19 lockdown in Hong Kong in May 2020, explained to us how the complication has intensified during the lockdown:

Yes, the internet has always been an issue. Sometimes our CBS students and their parents cannot access the school homepage and are therefore not informed. Depending on the schools and class levels, there are also homework to be done that requires the internet. Websites that we use in Hong Kong commonly like Googleare not accessible. That is a problem. … Now with the lockdown [since late January 2020] when we put everything online, it is even more problematic. Some CBS have been completely out of touch even since the border was closed. It is a big problem.

Understandably, many CBS parents are worried about their children being ‘left behind’ and excluded, as we observed in parents’ chatrooms and news media. The lockdown has excluded their children from attending school, both physically and digitally. The Hong Kong Government has been urged by the city’s legislators and social critics to address the widening digital divide (Lai, 2020; Legislative Council of the Hong Kong SAR Government, 2020). Necessary measures to improve the situation of under-privileged children have to go beyond the school premises to offer high-speed internet and computer access at home. Tackling CBS-specific digital challenges is additionally complex due to the transborder life of the children and the difference in digital governance on both sides of the border.

#### At the school gate

Finally, we focus on the role of the school as a space where identity politics and in/exclusion are contested among the parents. Parents and guardians seldom enter the schools in Hong Kong, but those with younger pupils often assemble regularly outside the school gate while dropping off or picking up the children. In our research context, the school gate is where “fleeting” or unintended encounters (Valentine, 2014; Ye, 2015), acquaintances or friendships, but also prejudices, can be observed. Here, we provide illustrations of the last of these—the aspect most commonly discussed by our interviewees. Their perceptions should be understood in the context of a commonly expressed negative attitude, prevalent among the general Hong Kong public, against the Mainland Chinese population (Equal Opportunities Commission, 2019). Cross-border families, in particular, are often represented as needy receivers of social services, or affluent and voracious people preying local resources from real estate to baby formula.

An incident that happened a few years ago remains in the collective memory of CBS families. For the 2013–14 school year, the Education Bureau announced that there were 1400 more applicants than school spaces available in North District (Pak, 2013). This showed serious planning failure of the Hong Kong government, as birth (including those by Mainland mothers) data had been available for planning school placements for these Hong Kong children. This “surprise” announcement aroused understandable worries among Hong Kong parents in the District about “losing the competition from the outsiders” for a desirable school place. A small number of Hong Kong parents staged protests at various schools to express their concern on the day when parents received their admission results. These few and small-scale actions drew an obvious the division between the “true local” and cross-border families. Discriminative vocabularies such as “Go back to the Mainland, locust children!” were used by parents with more extreme “nativists” views (Fung, 2015). The high level of political sensitiveness was reflected by the heavy police presence at the “popular” schools in North District on the day when school place allocation was announced.

While such high-level contestations at the school gate have been rare, identity politics are played out in more or less subtle manner on a daily basis. SY (Mainland mother of two children, husband Hong Kong resident, interviewed in March 2018) underlines her feeling of not belonging , at the school gate:

I bring my son to school. There are always other [local] parents at the school entrance, but I don’t talk to them. We smile at each other, but that’s it. I don’t feel belonged..

ZYY, also a CBS mother (two children, “double not” family, interviewed in 2018) shares her experience of prejudice at the school gate, and on the bus:

Some Hong Kong locals reject us…. When they [local Hong Kong parents at the school gate] hear that we speak Mandarin, they discriminate against us … Even some (Hong Kong) bus-drivers, they have different attitudes toward Mainlanders.

In the digital era, a “school gate” also takes forms online as parent forums or chatrooms. Parents come together to share information and experiences, and to seek and give mutual support. SY continues to tell us about her lack of belonging also in the digital space:

I don’t feel belonged. Even in the school chatrooms, we talk about different things. We are just different.

Our interviewees’ sharing makes clear how the school is a dynamic and power-traversed space where social differences between the “locals” and the “others” are played out, most intensely along class and language lines. CBS parents are, however, not passive victims in these contestations. They organise their own resource networks, making use of common social media in the Mainland, to tackle issues that concern them in particular. Hence, they have built their own ‘school gates’ in the digital space. We conducted observation on two WeChat groups, one initiated by a CBS parent and another by a social service organisation. These digital “school gates” are exclusive space. In many ways, they serve as a safe, third space for CBS parents. The purpose of this exclusive space is, however, to facilitate their children’s integration into the “Hong Kong education” that they highly value. Here again, we see how the process of inclusion and exclusion should not be seen as simply opposites. On the contrary, they are relational, reproduce and redefine each other in dynamic ways.

## Conclusions

Focusing on the cross-border education phenomenon along the Shenzhen–Hong Kong border, our paper has substantiated our understanding of the role the school in migration and inclusion as a meso-level organisation, mediating macro-level institutional politics and micro-level lived experiences. Using a relational, spatial perspective, we analysed the role of school in (co)producing geographies of in/exclusion for Mainland resident children in Hong Kong.

In order to make sense of Hong Kong schools’ agendas when it comes to CBS, and the challenges faced and actions taken by schools and teachers in their work with these children, it is necessary to go beyond the immediate school and education field. Drawing on our different data sets, we have shown how the school is embedded horizontally in a dynamic education mobility industry, and vertically linking macro-level politics and policy fields (in immigration, public health and education) and micro-level life experiences.

We underline the importance of spatialities in understand the role of the school in (re)producing socio-cultural differences and mediating social in/exclusion. Zooming in on three sets of spaces at school, namely (i) the (physical) classroom and school grounds, (ii) the digital classroom, and (iii) the school gate, we brought forth the lived experiences of our interviewees. Their narratives show how the school, as a web of intersecting physical, social and digital spaces, (co)produces and gives meaning to translocal social imageries and practices. Through our close-up examination of these spaces, we show that differences—and the aim of integration through education—are in a fluid and dialectic relationship rather than opposites. Paradoxical as it might seem, by deviating from the assimilationist approach and condoning students’ use of Mandarin in the classroom within their “social bubble” on the school grounds, teachers and the schools engender a more welcoming learning and social environment that facilitates (or at least does not hinder) longer-term integration.

While centering on the school, we do not treat it as a coherent and spatially bound organisation (Waters, 2017). We have shown how teachers deal with daily dilemmas arising from the contradictory macro-level policy directions and demands on the ground. They make decisions and often act at their discretion. Through these daily negotiations, they refine this transborder education field. Similarly, other school personnel (e.g. school principals, board members, social workers, but also cleaners and tuck shop staff), parents and students also exercise their agency in (co)producing and contesting the complex and changing geographies of in/exclusion.

The situation in Hong Kong and Mainland China is, of course, dynamic. We have here provided some first insights into the impact of the COVID-19 pandemic on CBS. Due to border closure and the restrictions in access to specific websites, CBS have been disadvantaged compared to their local peers. Parallel to the pandemic, Hong Kong has also faced immense socio-political challenges and changes (and unrest) in recent years. Consequently, some CBS parents have voiced their intention to bring their children back to the Mainland for school (Quinn, 2019) (confirmed by Teacher A in a follow-up online interview in 2020)^3^ As the future unfolds, the importance of the school as an organisation in mediating mobilities and in/exclusion the transborder region will remain, at least for some years, while its role might change. As a transborder organisation and a series of nested spaces where important actors and relevant institutions intersect, the school will continue to articulate with wider socio-cultural, political-economic processes in constituting the ever-changing geographies of in/exclusion in and beyond education, straddling Hong Kong and Mainland China.

## Data Availability

The paper draws on our qualitative research. The data that support the findings of this study are available from the corresponding author upon reasonable request.

## Footnotes

1 The official term is “boundary” for the border between Hong Kong and Shenzhen. Since ‘border’ is used generally in the scholarship, the term is also used here to denote the administrative boundary between the two cities.

2 *Suzhi* is a central notion in social governance in contemporary China. Reference to *suzhi* justifies social and political hierarchies of all sorts, with those of ‘high’ *suzhi* being seen as deserving more income, power and status than those of ‘low’ *sushi*, who are blamed for their ‘failure’.

3 Since Beijing’s National Security Law for Hong Kong took effect on 30 June 2020 Hong Kong’s Education Bureau has announced new guidelines and curriculum. These changes may impact the different spaces of the school—and parents’ perception of CBS—discussed in this paper, in years to come.
